# Intelligent energy optimization in park-wide farming considering user’s preferences

**DOI:** 10.1038/s41598-021-00732-6

**Published:** 2021-11-04

**Authors:** Cheng Jiangzhou, Niu Zhiyou

**Affiliations:** College of Engineering, Huazhong Agricultural University, Wuhan, 430070 China

**Keywords:** Climate sciences, Energy science and technology, Engineering

## Abstract

With the development of park-level agricultural, agricultural production and household electricity fusion, it is of great significance to promote users to actively respond to power consumption plan based on their own habits. In this paper, a multi-objective household intelligent power consumption optimization model is proposed from two aspects of economy and comfort. Firstly, the operating constraints of interruptible loads and non-interruptible loads were established based on the working characteristics of various household appliances. Then, the expenditure model was constructed to take into account the electricity sales situation of surplus electricity generated by photovoltaic, and a three-layer index system quantifying the influence of user preference on comfort level was constructed. The preference coefficient was determined by analytic hierarchy process, which was used to construct the users’ comfort level model. Finally, the multi-objective particle swarm optimization algorithm was applied to obtain optimization results. Considering the seasonal difference, the simulation showed that this model minimized the expenditure and increased the comfort level during summer and winter by 26.0% and 27.5% respectively.

## Introduction

Facing the rapid growth of power demand and the development of Park-level Agricultural, the home energy management system (HEMS) has received extensive attention. The main goal of HEMS is to minimize the energy consumption in the home and ensure users’ comfort level^1^. On the other hand, demand-side management (DSM) aims to encourage users to improve the efficiency of electricity consumption by modifying pattern and time of electricity usage. However, due to the lack of research on users’ electricity consumption habits and corresponding response strategies, users are less active in participating and hardly plan the rationality of electricity usage timely^2^. Therefore, HEMS has increasingly become an important way to realize DSM. At the same time of ensuring users’ comfort level, it is the focus of current research to achieve optimal control of household energy and effectively reduce expenditure.

In literature, a variety of methods are applied to obtain the optimal load scheduling pattern in HEMS. The research in^3^ used smart home controller to determine the best time to run smart household appliances. The authors in^4^ optimized the energy consumption of residential buildings based on the using probabilities of various electrical appliances. In^5^, the authors solved the optimization problem by multi-objective genetic algorithm, and the load distribution of smart grid within 36 h was optimized to minimize the electricity cost. A smart home energy management model based on Dijkstra algorithm has been proposed in^6^ which effectively reduced users’ expenditure. The authors in^7,8^ also proposed a non-cooperative game theory method to reduce electricity cost. Genetic algorithm (GA) has been utilized in^9^ to minimize energy consumption in residential, commercial and industrial sectors. The authors in^10^ reduced users’ expenditure and dissatisfaction based on multi-objective evolutionary algorithm (EA). In^11^, the authors reduced cost and maximized users’ comfort level by using binary particle swarm optimization (BPSO) and cuckoo search. At present, most researches are based on various optimization algorithms to solve the optimal scheduling strategy under the flexible electricity price structure of the power grid, with the main purpose of achieving economic benefits.

At present, a series of researches on DSM technology have been carried out at home and abroad. For example, the research^12^ used the predictive building energy management system of the global model to manage household energy consumption, which effectively reduced the cost. The authors in^13^ optimized the ice storage energy in the dynamic real-time electricity price environment by using DSM algorithm. In^14^, the authors proposed a closed-loop PID control system to balance energy demand and power generation market in real time.

In the issue of home energy management, users’ comfort and satisfaction are of significant concern. Comfort was initially used to evaluate users’ satisfaction with the buildings, mainly including thermal comfort level, visual comfort level and indoor air quality^15^. The study in^16^ optimized thermal comfort level by combining renewable energy and energy storage units, and managed the demand side. The authors in^17^ have analyzed the importance of occupancy mode in improving energy efficiency and comfort. Ref.^18^ quantified the visual comfort level of living space by changing the structure of windows. In^19^, the authors proposed a daylight control strategy, which improved the visual comfort effectively. The researchers in^20^ quantified air quality in the buildings by the concentration of carbon dioxide. The authors in^21^ reduced the expenditure within the comfort constraints based on the renewable energy prediction algorithm. In^22^, the authors proposed a multi-objective optimization model considering the correlation and comfort level of household appliances, which fully considered the cooperation of household appliances. The authors in^23^ considered the degree of correlation between loads, but comfort level modeling was not involved. A hybrid energy collaborative optimal control strategy has been proposed in^24^, which took temperature as the only standard of measuring comfort. The authors in^25^ optimized the control of air conditioner considering users’ comfort level and electricity consumption cost. The study in^26^, provides a balanced energy saving and comfortable life through users’ convenience rate as well as thermal comfort level. The study in^27^ involved household power scheduling of electric vehicles, but few other intelligent appliances. At present, there are few researches on users’ comfort level of electricity consumption, and users’ electricity habits as well as the different preference for different appliances are often overlooked. It is easy to sacrifice comfort for the sake of economy, thus affecting the enthusiasm of users’ participation.

To sum up, considering that the comfort level is closely related to users’ own electricity habits, this paper evaluated user's comfort level based on the user's preferences. According to the fuzzy analytic hierarchy process (FAHP), the preference coefficients of each household appliance were determined by using duration, function and carbon footprint as criterion factors.

The multi-objective optimization model of intelligent power consumption was constructed to minimize the expenditure and give users the maximum comfort at the same time. Finally, the influence of seasonal difference and preference coefficient on electricity consumption strategy is verified by simulation experiments.

## Analysis of household loads

From the perspective of operation scheduling, household appliances can be divided into two categories: controllable load and uncontrollable load^28^. According to the use of household appliances, controllable load can be divided into interruptible load and non-interruptible load. Users will not arbitrarily change their periods of power consumption, because uncontrollable load is not involved in the operation scheduling due to its fixed time slot and power^29^.

### Non-interruptible Load

Uninterruptible load refers to the load that can meet the electricity demand within a specified time, has a fixed number of working hours and cannot be interrupted. Within its allowable working time range [*a*_T,*i*_, *b*_T,*i*_], users can shift their working time according to their own electricity usage. Non-interruptible load shall meet the following constraints:

1 $$x_{T,i} \left( t \right) = 0\quad t \notin \left[ {a_{T,i} ,b_{T,i} } \right]$$

2 $$\sum\limits_{{t = t_{1} }}^{{t_{1} + \left( {L_{i} - 1} \right)}} {x_{T,i} \left( t \right) \ge L_{i} \cdot \left| {x_{T,i} \left( t \right) - x_{T,i} \left( {t - 1} \right)} \right|}$$

where, *x*_T, *i*_(*t*) is the uninterruptible operation state of electrical appliance *i* at time *t*; *t*_1_ is the start time of non-interruptible load; *L*_*i*_ is the minimum number of consecutive working hours during the operation of an non-interruptible load *i*. Equation (1) represents the working state constraint of non-interruptible load *i* at time *t*. Equation (2) means the constraint that non-interruptible load means to operate from *t*_1_.

### Interruptible load

Interruptible load refers to the load that users can use interruptively according to their own electricity habits under the condition of keeping the total daily task quantity unchanged. Within its allowable working time range [*a*_I,i_, *b*_I,i_], electrical appliance *i* shall complete tasks specified by users before the latest allowable closing time *b*_I,*i*_, and the specific working time is not fixed. Interruptible load shall meet the following operating condition constraints:

3 $$x_{I,i} \left( t \right) = 0\quad t \notin \left[ {a_{I,i} ,b_{I,i} } \right]$$

In order to avoid irreparable damage to the electrical appliances caused by frequent start and stop ^30^, the start-stop duration of interruptible load shall meet the following constraints:

4 $$\sum\limits_{{t = t_{1} }}^{{t_{1} + L_{I,i} }} {x_{I,i} \left( t \right) = L_{I,i} \cdot x_{I,i} \left( t \right)}$$

where, *t*_1_ is the start time of non-interruptible load; *L*_I,__*i*_ is the number of time slots during interruptible load *i* starting and finishing work once.

## Intelligent power consumption optimization model

### Expenditure model

Assuming that the surplus electricity generated by photovoltaic (PV) can be sold to the power grid, the total electricity expenditure includes electricity purchase cost and sale cost. During operating time [*a*_I,i_, *b*_I,i_], expenditure model is established as follows:

5 $$\begin{aligned} \min C_{{{\text{total}}}} &= \sum\limits_{t = 1}^{24} {C_{b} \left( t \right) \cdot \sum\nolimits_{i \in A}^{{}} {\left[ {p_{i} \cdot x_{i} \left( t \right) \cdot \left( {1 - {\text{Sen}}\left( t \right)} \right)} \right]} } \hfill \\ &\quad- \sum\limits_{t = 1}^{24} {\left[ {G\left( t \right) - \sum\nolimits_{i \in A}^{{}} {\left( {p_{i} \cdot x_{i} \left( t \right)} \right) - M\left( t \right)} } \right] \cdot C_{s} \left( t \right) \cdot {\text{Sen}}\left( t \right)} \hfill \\ \end{aligned}$$

The number of operating periods and power limitation of each period for each electrical appliance shall meet the following constraints:

6 $$\forall i \in A,\sum\limits_{t = 1}^{24} {x_{i} \left( t \right) = N_{i} }$$

7 $$\forall t \in \left[ {1,24} \right],\sum\nolimits_{i \in A} {p_{i} \cdot x_{i} \left( t \right) + M\left( t \right) - G\left( t \right) \le D\left( t \right)}$$

8 $$x_{i} \left( t \right) = \left\{ \begin{gathered} 1\begin{array}{*{20}c} {} & {} \\ \end{array} {\text{appliance}} i{\text{ is working at time}} t \hfill \\ 0\begin{array}{*{20}c} {} & {} \\ \end{array} {\text{else}} \hfill \\ \end{gathered} \right.$$

9 $$S{\text{en}}\left( t \right) = \left\{ \begin{gathered} 1\begin{array}{*{20}c} {} & {} \\ \end{array} \sum\nolimits_{i \in A} {p_{i} \cdot x_{i} \left( t \right) + M\left( t \right) - G\left( t \right) \le 0} \hfill \\ 0\begin{array}{*{20}c} {} & {} \\ \end{array} {\text{else}} \hfill \\ \end{gathered} \right.$$

where, *C*_total_ is total electricity expenditure; *A* is a collection of all the household appliances; *C*_b_ and *C*_s_ are power purchase price and selling price respectively; *p*_*i*_(*t*) is working power of electrical appliance at time *t*; *Sen*(*t*) indicates weather there is any electricity remaining at time *t*, *Sen*(*t*) = 1 means selling electricity to the power grid, *Sen*(*t*) = 0 means purchasing electricity from the power grid; *G*(*t*) is the expected power generated by PV; *M*(*t*) is the power that must be consumed; *D*(*t*) is the power upper limit; *N*_*i*_ is the number of cumulative running periods.

### Comfort level model

The formulation of electricity consumption plan is influenced by the load characteristics and user preferences of various electrical appliances. Preference coefficient *w* is introduced to build a comfort level model for describing the influence of the change degree of power consumption plan on users^22^, as shown in (10). The smaller the change of the users’ electricity plan before and after optimization, the more comfortable the electricity plan after optimization is.

10 $$\max S = 1 - \frac{{\sum\nolimits_{i \in A} {w_{i} \cdot p_{i} \left( t \right)\sum\limits_{t = 1}^{24} { \cdot \left| {x_{i}^{0} \left( t \right) - x_{i} \left( t \right)} \right|} } }}{{\sum\nolimits_{i \in A} {\sum\limits_{t = 1}^{24} {p_{i} \left( t \right) \cdot x_{i}^{0} \left( t \right)} } }}$$

where, *S* is users’ comfort level; *x*^0^_*i*_(*t*) is the original power plan of controllable electrical appliances *i* at time *t*; *x*_*i*_(*t*) is the power consumption plan of the optimized controllable electric appliance *i* at time *t*.

If the daily total task amount of household appliances is consistent before and after optimization, there is

11 $$\sum\limits_{t = 1}^{24} {x_{i} \left( t \right) = } \sum\limits_{t = 1}^{24} {x_{i}^{0} \left( t \right)}$$

### Determination of preference coefficient

Under the limitations of coat and energy, users need to decide the scheduling priority order of appliances based on their own preferences. In order to correctly identify users' preferences and quantify the effect of objective information and electricity environment on users’ comfort level, the preference coefficient of different electrical appliances is determined by using fuzzy hierarchy process^31,32^. The rising of *w* shows that users further expect the time slot of the appliance would stay stable, and the decreasing of *w* shows the increasing flexibility of the time slot of the appliance.

#### Construction of fuzzy hierarchy model

**Figure 1:**
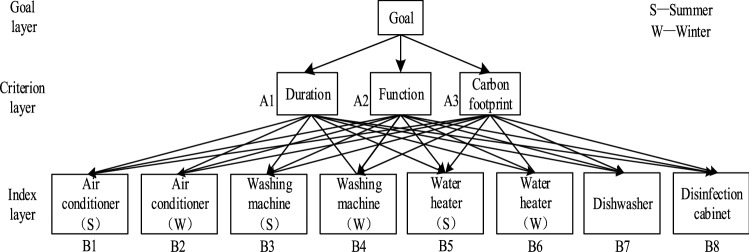
Fuzzy hierarchical diagram.

The structural model is the basis of FAHP. It decomposes complex problems into several elements, and then divides these elements into corresponding attributes according to their respective attributes, forming different levels. The degree of users' preference for all kinds of household appliances was taken as target G, and duration, function and carbon footprint were taken as the criterion layer A1, A2 and A3. Five typical household appliances were selected as the index layer B1-B8, where the seasonal difference of air conditioner, washing machine and water heater was taken into account. The fuzzy hierarchy structure is shown in Fig. 1.

#### Construction of fuzzy complementary judgment matrix

For the lower element $\mathrm{X}=[{\mathrm{x}}_{1},{\mathrm{x}}_{2},\cdots ,{\mathrm{x}}_{\mathrm{n}}]$ $\mathrm{X}=[{\mathrm{x}}_{1},{\mathrm{x}}_{2},\cdots ,{\mathrm{x}}_{\mathrm{n}}]$ set belonging to the same element in the hierarchical structure model, users compare the elements in X in pairs. According to the meaning of each fuzzy scale listed in Table 1, the relative importance of the two elements is quantitatively expressed with the fuzzy scale to generate fuzzy judgment matrix R.

**Table 1:** 1–9 scale scoring rules.

Score	Explanation
1	Both are equally important
3	The former is slightly more important than the latter
5	The former is more important than the latte
7	The former is much more important than the latter
9	The former is more dominant than the latter
2, 4, 6, 8	Intermediate score between 1, 3, 5, 7 and 9

Since the triangular fuzzy number ($\mathrm{l},\mathrm{ m},\mathrm{ u}$ $\mathrm{l},\mathrm{m},\mathrm{u}$) can better reflect the judgment of the relative importance of the two elements by subjective evaluators, this paper uses the triangular fuzzy number ${\mathrm{r}}_{\mathrm{ij}}$ ${\mathrm{r}}_{\mathrm{ij}}$ to express the relative importance of the element $\mathrm{i}$ $\mathrm{i}$ and $\mathrm{j}$ $\mathrm{j}$, and constructs a pairwise comparison fuzzy judgment matrix.

12 $$R_{n \times n} = \left[ {\begin{array}{*{20}c} {r_{11} } & \cdots & {r_{1n} } \\ \vdots & \ddots & \vdots \\ {r_{n1} } & \cdots & {r_{nn} } \\ \end{array} } \right]$$

13 $$r_{ij} = \left( {l_{ij} ,m_{ij} ,u_{ij} } \right), r_{ii} = 1$$

14 $$r_{ji} = r_{ij}^{ - 1} = \left( {{\raise0.7ex\hbox{$1$} \!\mathord{\left/ {\vphantom {1 {u_{ij} }}}\right.\kern-\nulldelimiterspace} \!\lower0.7ex\hbox{${u_{ij} }$}},{\raise0.7ex\hbox{$1$} \!\mathord{\left/ {\vphantom {1 {m_{ij} }}}\right.\kern-\nulldelimiterspace} \!\lower0.7ex\hbox{${m_{ij} }$}},{\raise0.7ex\hbox{$1$} \!\mathord{\left/ {\vphantom {1 {l_{ij} }}}\right.\kern-\nulldelimiterspace} \!\lower0.7ex\hbox{${l_{ij} }$}}} \right)$$

In the formula (13), (14): $\mathrm{i},\mathrm{j}=\mathrm{1,2},\cdots .\mathrm{n}$ $\mathrm{i},\mathrm{j}=\mathrm{1,2},\cdots .\mathrm{n}$; ${\mathrm{l}}_{\mathrm{ij}}$ ${\mathrm{l}}_{\mathrm{ij}}$ and ${\mathrm{u}}_{\mathrm{ij}}$ ${\mathrm{u}}_{\mathrm{ij}}$ are the upper and lower bounds, which represent the most conservative and optimistic evaluation, and $m_{ij}$ ${\mathrm{m}}_{\mathrm{ij}}$ ${\mathrm{m}}_{\mathrm{ij}}$ is the median value, which represents the most likely importance of the relationship between the two elements.

As the construction of judgment matrix is affected by subjective factors, the matrix cannot be completely consistent due to the errors in users' electricity habits and scale, so it is necessary to conduct consistency test for each judgment matrix to avoid the occurrence of large errors. Consistency index, random consistency index and consistency ratio were calculated as shown in Eq. (15)–(17), and the elements in the judgment matrix were adjusted until the consistency ratio was less than 10%.

15 $$CI = \frac{{\lambda_{\max } - n}}{n - 1}$$

16 $$RI = 1.98\left( {\frac{n - 2}{n}} \right)$$

17 $$CR = \frac{CI}{{CR}} < 10\%$$

where, *n* is the number of indicators; *λ*_max_ is maximum eigenvalue; *CI* is the consistency index; *RI* is the random consistency index; *CR* is the consistency ratio.

#### Hierarchical single sorting, calculating single-layer fuzzy weight

1. Construct a matrix of fuzzy evaluation factors E:
  18 $$E = (e_{ij} )_{n \times n} = \left[ {\begin{array}{*{20}c} 1 & {1 - \frac{{\mu_{12} - l_{12} }}{{2m_{12} }}} & \cdots & {1 - \frac{{\mu_{1n} - l_{1n} }}{{2m_{1n} }}} \\ {1 - \frac{{\mu_{21} - l_{21} }}{{2m_{21} }}} & 1 & \cdots & {1 - \frac{{\mu_{2n} - l_{2n} }}{{2m_{2n} }}} \\ \vdots & \vdots & \vdots & \vdots \\ {1 - \frac{{\mu_{n1} - l_{n1} }}{{2m_{n1} }}} & {1 - \frac{{\mu_{n2} - l_{n2} }}{{2m_{n2} }}} & \cdots & 1 \\ \end{array} } \right]$$
  In the formula (18), $\frac{({\mathrm{u}}_{\mathrm{ij}}-{\mathrm{l}}_{\mathrm{ij}})}{2{\mathrm{m}}_{\mathrm{ij}}}$ $\frac{({\mathrm{u}}_{\mathrm{ij}}-{\mathrm{l}}_{\mathrm{ij}})}{2{\mathrm{m}}_{\mathrm{ij}}}$ is called the standard deviation rate, which is used to measure the degree of ambiguity of the expert’s assessment of the importance of the element. The more clearly the judgment, the higher the credibility of the evaluation result.
2. Calculate and adjust the judgment matrix T:
  19 $$T = M \times E = \left[ {\begin{array}{*{20}c} {m_{11} } & \cdots & {m_{1n} } \\ \vdots & \ddots & \vdots \\ {m_{n1} } & \cdots & {m_{nn} } \\ \end{array} } \right] \times \left[ {\begin{array}{*{20}c} 1 & \cdots & {1 - \frac{{u_{1n} - l_{1n} }}{{2m_{1n} }}} \\ \vdots & \ddots & \vdots \\ {1 - \frac{{u_{n1} - l_{n1} }}{{2m_{n1} }}} & \cdots & 1 \\ \end{array} } \right]$$
  In the formula (19), each element of the M matrix is composed of triangular fuzzy numbers ${\mathrm{m}}_{\mathrm{ij}}$ ${\mathrm{m}}_{\mathrm{ij}}$, which is defined as a median matrix.
3. Divide each column in the adjusted judgment matrix $\mathrm{T}$ $\mathrm{T}$ by ${\mathrm{T}}_{\mathrm{ii}}$ ${\mathrm{T}}_{\mathrm{ii}}$, construct a new matrix with a diagonal of 1, and define it as the judgment matrix Q.
4. The square root method is used to calculate the weight of each indicator.

First calculate the nth root of all elements in each row of the judgment matrix Q:

20 $$w_{i}^{^{\prime}} = \left( {\prod\limits_{j = 1}^{n} {d_{ij} } } \right)^{\frac{1}{n}} ,i = 1,2, \cdots ,n$$

Then, normalize the indicator ${w}_{i}$:

21 $$w_{i} = \frac{{w_{i}^{^{\prime}} }}{{\sum\limits_{i = 1}^{n} {w_{i} } }},i = 1,2, \cdots ,n$$

Obtain $\mathrm{W}={[{\mathrm{w}}_{1},{\mathrm{w}}_{2},\cdots ,{\mathrm{w}}_{\mathrm{n}}]}^{\mathrm{T}}$ $\mathrm{W}={[{\mathrm{w}}_{1},{\mathrm{w}}_{2},\cdots ,{\mathrm{w}}_{\mathrm{n}}]}^{\mathrm{T}}$ as the approximate value of the required weight.

#### Level total ranking; calculate the comprehensive weight of each factor to the target level

According to the hierarchical structure model, apart from the target layer, from high to low, according to the single ordering of this layer and the total ordering of the upper layer, the weight of the influence of the elements of each layer on the target problem can be obtained.

Assuming that there are m elements in the s-th layer ${\mathrm{A}}_{1},{\mathrm{A}}_{2},\cdots {\mathrm{A}}_{\mathrm{m}}$ ${\mathrm{A}}_{1},{\mathrm{A}}_{2},\cdots {\mathrm{A}}_{\mathrm{m}}$, the corresponding level is always sorted as follows ${\mathrm{a}}_{1},{\mathrm{a}}_{2},\cdots ,{\mathrm{a}}_{\mathrm{m}}$ ${\mathrm{a}}_{1},{\mathrm{a}}_{2},\cdots ,{\mathrm{a}}_{\mathrm{m}}$; the $(\mathrm{s}+1)(\mathrm{s}+1)$-th layer has n elements ${\mathrm{B}}_{1},{\mathrm{B}}_{2},\cdots ,{\mathrm{B}}_{\mathrm{n}}$ ${\mathrm{B}}_{1},{\mathrm{B}}_{2},\cdots ,{\mathrm{B}}_{\mathrm{n}}$, and the single-level ordering of an element in the upper e-level is as follows:${\mathrm{b}}_{1\mathrm{i}},{\mathrm{b}}_{2\mathrm{i}},\cdots ,{\mathrm{b}}_{\mathrm{ni}}$ ${\mathrm{b}}_{1\mathrm{i}},{\mathrm{b}}_{2\mathrm{i}},\cdots ,{\mathrm{b}}_{\mathrm{ni}}$, Set the corresponding weights that are not dominated by the element $\mathrm{Ai}$ $\mathrm{Ai}$ to 0; therefore, the total order of the $\left(\mathrm{s}+1\right)$ $\left(\mathrm{s}+1\right)$-th level elements ${\mathrm{B}}_{\mathrm{i}}$ ${\mathrm{B}}_{\mathrm{i}}$ to the target problem is:

22 $$b_{i} = \sum\limits_{j = 1}^{m} {a_{j} b_{ij} ,i = 1,2, \cdots ,n}$$

The comprehensive weight is shown in Table 2.

**Table 2:** Results of index weight.

First index	Weight vector	Second index	Weight matrix	Comprehensive weight
A1 A2 A3	$$\left[ {\begin{array}{*{20}c} {0.283} \\ {0.074} \\ {0.643} \\ \end{array} } \right]$$	B1	$$\left[ {\begin{array}{*{20}l} {0.216} \hfill & {3.007} \hfill & {2.220} \hfill \\ {0.053} \hfill & {0.716} \hfill & {0.386} \hfill \\ {0.027} \hfill & {0.438} \hfill & {0.576} \hfill \\ {0.087} \hfill & {1.426} \hfill & {1.527} \hfill \\ {0.141} \hfill & {0.469} \hfill & {1.709} \hfill \\ {0.397} \hfill & {2.337} \hfill & {2.102} \hfill \\ {0.027} \hfill & {0.498} \hfill & {2.052} \hfill \\ {0.055} \hfill & {0.682} \hfill & {2.764} \hfill \\ \end{array} } \right]$$	0.424
		B2		0.087
		B3		0.060
		B4		0.193
		B5		0.153
		B6		0.416
		B7		0.129
		B8		0.173

## model solution

**Figure 2:**
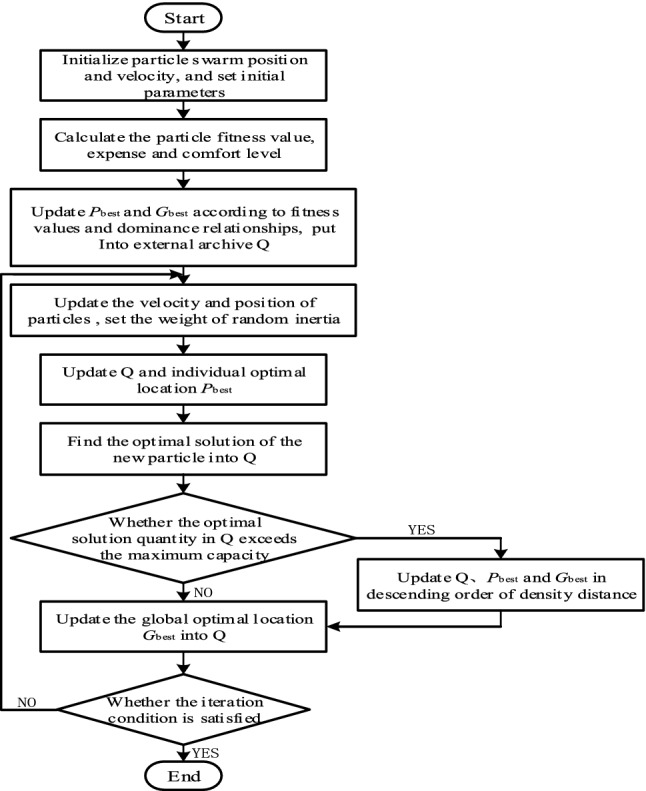
Flowchart of multi-objective particle swarm optimization.

In practice, household intelligent power consumption optimization needs to consider economy and comfort comprehensively. The traditional penalty function method transforms multi-objective optimization into single-objective optimization, which requires repeatedly setting the value of the calibration weight and has low operating efficiency. Therefore, particle swarm optimization algorithm based on random weights was used to solve the multi-objective problem. However, the main problem of multi-objective optimization is that it is difficult to achieve multiple optimization goals at the same time, and the optimization of one goal may hinder the realization of other optimization goals. Therefore, pareto mechanism is used in this paper to find a compromise solution to measure multiple goals. The optimal location is stored by external documents, and the diversity of external documents is ensured by filtering and deleting non-dominant solutions. Pareto solutions of relatively sparse regions are used as global guides to ensure the convergence and diversity of the algorithm.as shown in Fig. 2.

1. Set initial parameters, such as population size *S*, particle dimension *i*, etc., and each particle corresponds to the scheduling plan of household appliances;
2. Initialize a group of random particles, give the initial speed and position, and input the basic information of each appliance, electricity price and power information of each time period;
3. Calculate the fitness value according to the objective function (5) and (10);
4. Compare fitness value of particles, update the individual optimal location *P*_best_ and non-inferior solution set according to the domination relation^33^, randomly select the global optimal position *G*_best_ from the non-inferior solution set and put it into external archive Q;
5. Set random weights according to (23). The influence of particle historical velocity on current velocity is random^34^, and the non-convergence caused by linear decline of *w* is avoided to some extent;
  23 $$\left\{ \begin{gathered} w = \mu + \sigma \cdot N\left( {01} \right) \hfill \\ \mu = \mu_{\min } + \left( {\mu_{\max } - \mu_{\min } } \right) \cdot rand\left( {01} \right) \hfill \\ \end{gathered} \right.$$
  where, *w* is inertia weight; *µ* is the average of random weights; *σ* is variance of random weights; *N*(0,1) is random numbers of standard normal distribution.
6. Update the particle velocity and position according to (24) and (25);
  24 $$\begin{aligned} V_{i} \left( {t + 1} \right) & = wV_{i} \left( t \right) + c_{1} r_{1} \left( {P_{{{\text{best}}}} - X_{i} \left( t \right)} \right) \\ & \quad + c_{2} r_{2} \left( {G_{{{\text{best}}}} - X_{i} \left( t \right)} \right) \\ \end{aligned}$$
  where, *w* is inertia weight; *c*_1_ and *c*_2_ are learning factors; *r*_1_ and *r*_2_ are random numbers between 0 and 1; *V*_i_(t) and *X*_i_(t) are respectively the velocity and position of particle *i* in time period *t*.
  25 $$X_{i} \left( {t + 1} \right) = X_{i} \left( t \right) + V_{i} \left( {t + 1} \right)$$
7. Recalculate fitness values, and update *P*_best_ and external archive Q;
8. Update the optimal solution and put it into Q, judge whether the optimal solution quantity in Q exceeds the maximum capacity Q_max_. If it exceeds, find *G*_best_ by descending cropping in (26);
  26 $$D\left( {X_{i} } \right) = \frac{{\sum\limits_{N = 1}^{2} {\left| {f_{N} \left( {X_{j} } \right) - f_{N} \left( {X_{k} } \right)} \right|} }}{{f_{\max } }}$$
  where, *f*_*N*_(*X*_*i*_) is *N* objective function value of *X*_*i*_; *f*_max_ is the maximum value in the external document.
9. Judge whether the maximum number of iterations *T*_max_ is reached. If not, skip to step 5 to continue the iteration; if so, end the cycle and output the optimal opening period and optimization results of each appliance.

## Example simulation and analysis

### Parameter setting

Five typical controllable electrical appliances in a family were selected for experiments. One day was divided into 24 time slots, and it assumed that some electrical appliances whose working hours did not reach 1 h were calculated as 1 h. Specific parameters of each appliance^30^ are shown in Table 3.

**Table 3:** Related information of household appliances.

Parameter	Interruptible load		Non-interruptible load			
	Air conditioner	Water heater	Washing machine		Dishwasher	Disinfection cabinet
*p*_*i*_	1.20	1.80	0.75	1.00		0.52
[*a*_*i*_, *b*_*i*_]	11–23	5–24	9–21	8–21		18–22
*N*_*i*_ (S)	5	4	2	2		1
*N*_*i*_(W)	2	7	4	2		1
*L*_*i*_	1	1	2	1		1
*W*_*i*_(S)	0.424	0.060	0.153	0.173		0.129
*W*_*i*_(W)	0.087	0.193	0.416			

According to TOU, the purchase price, sale price and power upper limit were obtained from the power company. The estimated power generated by PV was obtained according to the historical data and weather forecast, and the necessary operating power was given by the users. Specific price and power data of each time slot^35^ are shown in Table 4.Where, *C*_b_ and *C*_s_ are power purchase price and selling price respectively; *D*(*t*) is the power upper limit; *G*(*t*) is the expected power generated by PV; *M*(*t*) is the power that must be consumed.

**Table 4:** Time-related information.

Time slot	*C*_b_(yuan)	*C*_s_(yuan)	*D*(S/W)(kW)	*G*(S/W)(kW)	*M*(kW)
1	0.44	0.52	3/4	0.00	0.40
2	0.44	0.52	3/4	0.00	0.30
3	0.44	0.52	3/4	0.00	0.20
4	0.44	0.52	3/4	0.00	0.30
5	0.44	0.52	3/4	0.00	0.20
6	0.44	0.52	3/4	0.00	0.00
7	0.59	0.52	3/4	0.01/0.00	0.00
8	0.59	0.52	3/4	0.07/0.00	0.20
9	0.59	0.52	3/4	0.21/0.01	0.30
10	0.59	0.52	3/4	0.32/0.09	0.20
11	0.59	0.52	3/4	0.47/0.30	0.25
12	0.59	0.52	3/4	0.86/0.63	0.30
13	0.53	0.52	3/4	0.92/0.47	0.30
14	0.53	0.52	3/4	0.98/0.50	0.30
15	0.53	0.52	3/4	0.61/0.21	0.25
16	0.53	0.52	3/4	0.38/0.19	0.25
17	0.53	0.52	3/4	0.14/0.02	0.25
18	0.53	0.52	3/4	0.09/0.00	0.20
19	0.59	0.52	3/4	0.01/0.00	0.30
20	0.59	0.52	3/4	0.00	0.30
21	0.59	0.52	3/4	0.00	0.30
22	0.59	0.52	3/4	0.00	0.30
23	0.59	0.52	3/4	0.00	0.20
24	0.59	0.52	3/4	0.00	0.25

Due to the complicated calculation of this problem, the population number *S* is set to 100; *c*_1_ and *c*_2_ are learning factors. If it is too small, the particles may be far away from the target area, if it is too large, it will lead to flying over the target area, generally *c*_1_ = *c*_2_ = 2; μ_max_ and *μ*_min_ are random numbers on [0,1], which are set to 0.8 and 0.5 in this paper. The variance σ and the number of iterations *T*_max_ affect the accuracy of the algorithm. Considering the results of multi-objective optimization, this paper is set to 0.2 and 150 respectively. The simulation parameters were set as shown in Table 5.

**Table 5:** Simulation parameter setting.

Parameter	Value	Parameter	Value
*S*	100	*σ*	0.2
*i*	5	*μ*_max_	0.8
*c*_1_, *c*_2_	2	*μ*_min_	0.5
*T*_*max*_	150	*Q*_max_	100

### The analysis of optimization results

Considering seasonal difference, the household intelligent power consumption behavior was optimized and analyzed in three different situations.

**Figure 3:**
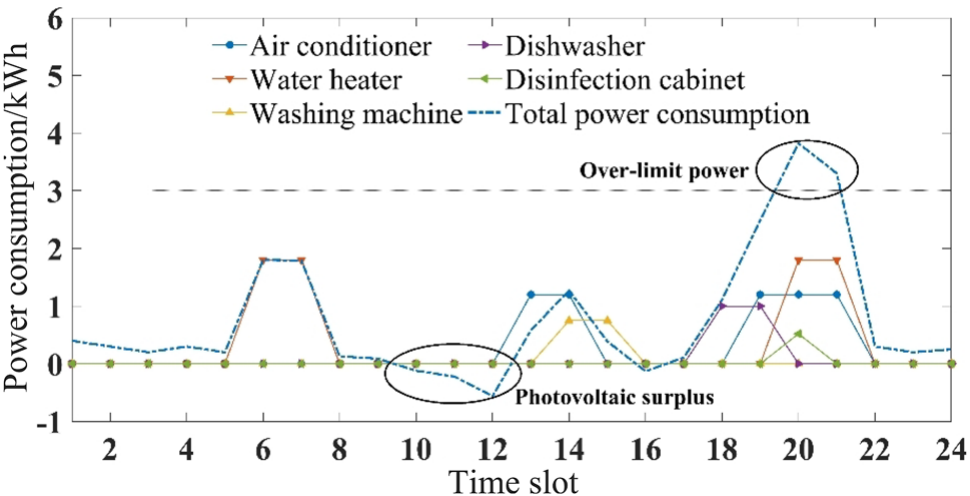
Original power consumption plan in summer.

**Figure 4:**
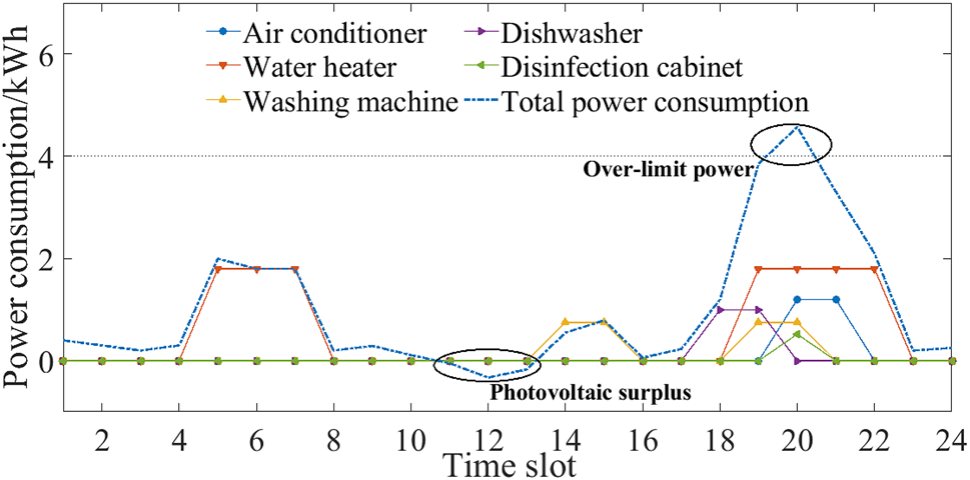
Original power consumption plan in winter.

**Figure 5:**
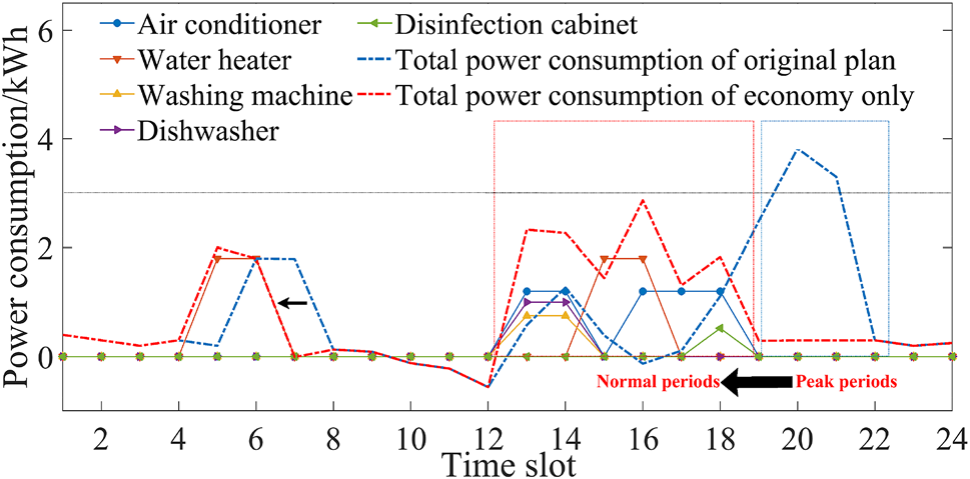
Power consumption plan aiming to minimize costs in summer.

**Figure 6:**
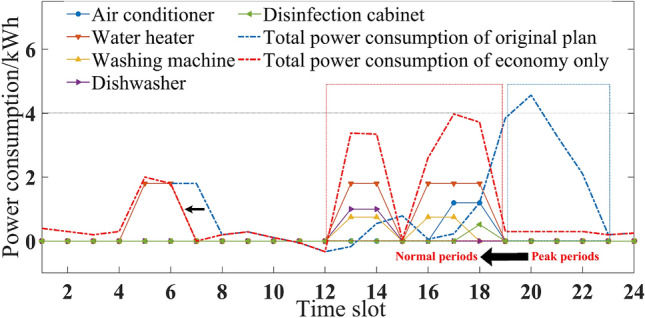
Power consumption plan aiming to minimize costs in winter.

**Figure 7:**
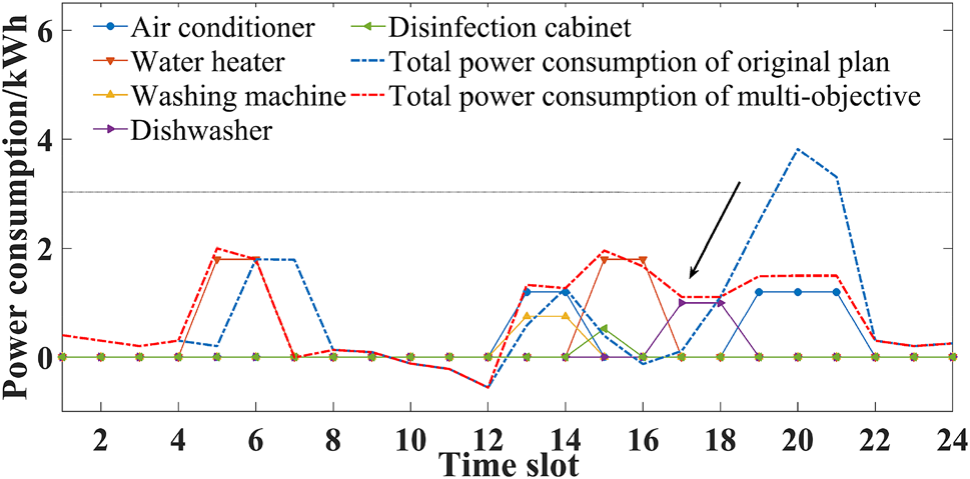
Power consumption plan of multi-objective optimization in summer.

**Figure 8:**
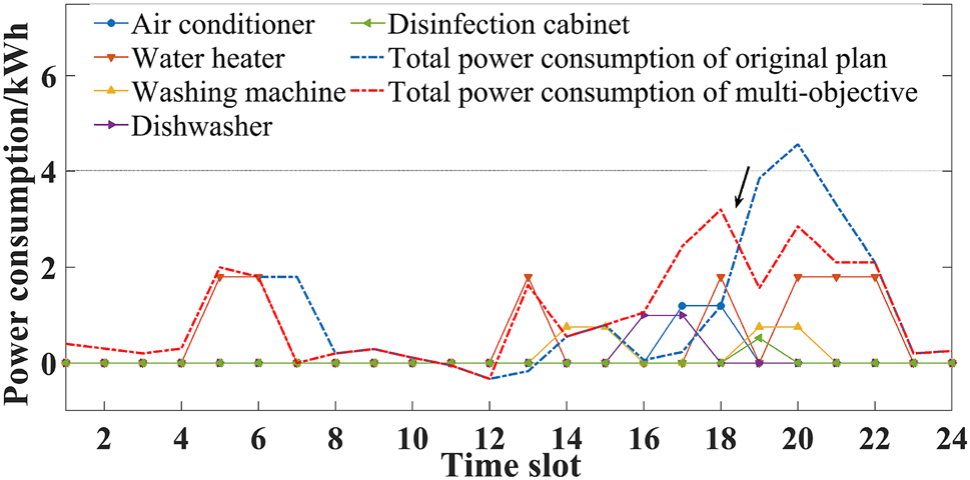
Power consumption plan of multi-objective optimization in winter.

1. If comfort was the only consideration, the original power consumption plan of five household appliances is shown in Figs. 3 and 4. Users’ power consumption habits did not change and the highest comfort level was 1. The use of appliances was mainly concentrated in the peak period, when the power over the limit, and the highest cost. In comparison with Figs. 3 and 4, there were significant seasonal differences between the duration and time slot in the use of air conditioner, water heater and washing machine. For example, users were more rely on the use of air conditioner in summer, and its daily operating duration was 5 h, which was more than 2 h in winter.
2. If expenditure was the only consideration, the power consumption plan is shown in Figs. 5 and 6. At this time, the expenditure was the lowest. Compared with the original power consumption plan; the expenditure in summer and winter decreased by 41.71 points and 86.69 points respectively. The comparison of Figs. 3 and 4 showed that users' power consumption habits were changed greatly. In order to minimize expenditure, the slots of using household appliances were adjusted from peak periods to normal periods of TOU. Although the expenditure is significantly reduced, too much adjustment of operating period leads to the worst users’ comfort without real optimization effect.
3. The multi-objective optimal power consumption plan is shown in Figs. 7 and 8 after introducing the preference coefficient. In summer, air conditioner had the highest preference coefficient. According to the comparison between Figs. 7 and 3, in order to ensure comfort level, the operating periods were not adjusted, and the comfort level was increased by 26.0% compared with the consideration of economy only. The water heater with lowest preference coefficient adjusted its partial time slots from peak periods to normal periods. For example, the expenditure reduced 20.11 points compared with the original plan when the operation of the 7th period was advanced to the 5th period. In winter, washing machine had the highest preference coefficient. According to the comparison between Figs. 8 and 4, the operating periods of the washing machine were not adjusted, and the comfort level was increased by 27.5% compared with the consideration of economy only. For air conditioner with the lowest preference coefficient, the operating periods were adjusted from peak periods to normal periods, and the expenditure was reduced by 42.17 points. It can be seen that multi-objective optimization not only guarantees users’ comfort level, but also minimizes the expenditure. It effectively alleviates the peak power consumption pressure and avoids the occurrence of over-limit power.

### The influence of preference coefficient

**Figure 9:**
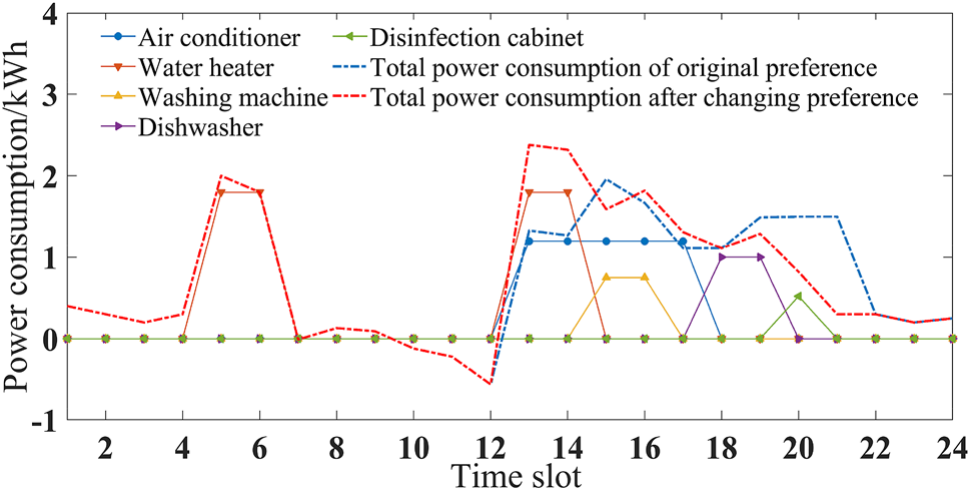
Power consumption plan after changing preference coefficient in summer.

Different users have different degrees of preference for various electrical appliances due to their differences in electricity consumption habits. In order to verify the influence of the introduction of preference coefficients on the results of multi-objective electricity optimization, the analytic hierarchy process was used to recalculate the changed preference coefficients, as shown in Table 6, and the multi-objective optimization results obtained after the change are shown in Fig. 9.

**Table 6:** Preference coefficient in summer.

		Air conditioner	Water heater	Washing machine	Dishwasher	Disinfection cabinet
*w*_*i*_(S)		0.191	0.142	0.085	0.274	0.303

Compared with Figs. 3, 7 and 9, it was obvious that with the change of preference coefficient, power consumption plan also changed greatly. By comparing Tables 3 and 6, it could be seen that the preference coefficient of the air conditioner changed from 0.424 to 0.191, which meant that the constraint restricting the change of its power consumption plan became smaller. Its three operating periods were all adjusted from peak periods to normal periods. The preference coefficient of the dishwasher changed from 0.129 to 0.274, which meant that the constraint became larger and its operating periods were not adjusted. Compared with preference case 1, comfort level decreased by 10.9%, but expenditure also decreased by 21.28 points.

To sum up, results of specific expenditure and comfort level were obtained in all cases, as shown in Table 7. If only the realization of single objective optimization is considered, other objectives are often sacrificed^36,37^. In order to achieve economic benefits, the comfort of users' power consumption is easily neglected. Therefore, multi-objective optimization should be carried out to fully consider the impact of user preferences on comfort level^38^. The optimal power consumption plan is obtained, which can improve users’ comfort level while reducing the expenditure.

**Table 7:** Optimization results in different cases.

Optimization conditions		Summer		Winter	
		*C*_total_/points	*S*	*C*_total_/points	*S*
Original power plan		962.78	1.00	1324.92	1.00
Economy only		921.07	0.73	1238.23	0.69
**Multi-objective**	Preference case 1	942.67	0.92	1282.75	0.88
	Preference case 2	921.39	0.82		

In life, users can use the analytic hierarchy process to recalculate the changed preference coefficient based on their preference for time comfort, temperature comfort, electricity consumption habits, and real-time electricity prices, and obtain the changed multiple goals. The optimization results make home energy management optimization more accurate and practical^39–42^.

## Conclusion

In this paper, an intelligent household power consumption optimization model was established. Considering the surplus electricity generated by PV selling to the grid, it could reduce the expenditure to the maximum while ensuring users’ comfort level. The hierarchical analysis structure was constructed by combining duration, function and carbon footprint, which quantified the influence of users’ preference on comfort level. Considering the difference of seasonal electricity consumption, the influence of preference coefficient on multi-objective optimization results was verified by experimental comparison. Users can adjust preference coefficient based on their own habits. In the future, the optimal charging and discharging control of electric vehicles will be added to HEMS, and the users’ uncertain power consumption behavior will be further studied.
